# CcRR5 interacts with CcRR14 and CcSnRK2s to regulate the root development in citrus

**DOI:** 10.3389/fpls.2023.1170825

**Published:** 2023-04-17

**Authors:** Manman Zhang, Fusheng Wang, Zhou Hu, Xiaoli Wang, Qian Yi, Jipeng Feng, Xiaochun Zhao, Shiping Zhu

**Affiliations:** ^1^ Citrus Research Institute, Southwest University, Chongqing, China; ^2^ National Citrus Engineering Research Center, Southwest University, Chongqing, China

**Keywords:** citrus, response regulators, SnRK2s, root development, abiotic stresses

## Abstract

Response regulator (RR) is an important component of the cytokinin (CK) signal transduction system associated with root development and stress resistance in model plants. However, the function of *RR* gene and the molecular mechanism on regulating the root development in woody plants such as citrus remain unclear. Here, we demonstrate that *CcRR5*, a member of the type A RR, regulates the morphogenesis of root through interacting with CcRR14 and CcSnRK2s in citrus. *CcRR5* is mainly expressed in root tips and young leaves. The activity of *CcRR5* promoter enhanced by CcRR14 was proved with transient expression assay. Seven SnRK2 family members with highly conserved domains were identified in citrus. Among them, CcSnRK2.3, CcSnRK2.6, CcSnRK2.7, and CcSnRK2.8 can interact with CcRR5 and CcRR14. Phenotypic analysis of *CcRR5* overexpressed transgenic citrus plants indicated that the transcription level of *CcRR5* was associated with root length and lateral root numbers. This was also correlated to the expression of root-related genes and thus confirmed that *CcRR5* is involved in the root development. Taken together, the results of this study indicate that *CcRR5* is a positive regulator of root growth and CcRR14 directly regulates the expression of *CcRR5*. Both CcRR5 and CcRR14 can interact with CcSnRK2s.

## Introduction

Root system is an important organ for plant anchorage, support, absorption, and stress resistance (Zhuang et al., 2021). Plants initially perceive various stresses through their roots, modulating root system architecture through cell division, elongation, and differentiation to fight for chances to survive (Gupta et al., 2020). Plant hormones are known to affect root growth and development (Cai et al., 2022; Jia et al., 2022; Jiu et al., 2022). Phytohormones such as auxin and brassinosteroids (BRs) promote the elongation of primary root and initiation of lateral root (Yu et al., 2015; Wei and Li, 2016; Li et al., 2020b; Liu et al., 2021), whereas abscisic acid (ABA), cytokinin (CK), and jasmonic acid (JA) inhibit the root growth (Yang et al., 2017a; Deng et al., 2022; Liu et al., 2022). In general, CK is considered as a vital factor of root plasticity, as it not only influences root growth and development but also plays important roles in regulating the response to abiotic stress of root (Ha et al., 2012; Li et al., 2022).

In plants, CK signaling is mediated by a typical two-component system (TCS), which consists of histidine kinases (HKs), histidine phosphotransfer proteins (HPs), and response regulators (RRs) (Liang et al., 2012). RRs play a bridging role in receiving upstream phosphorylation signals and regulating the expression of downstream target genes (Yan et al., 2021). In *Arabidopsis thaliana*, *ARR1* and *ARR12* are involved in root plasticity development under low temperature by reducing the accumulation of auxin in roots (Zhu et al., 2015). *ARR7* and *ARR15* are essential to maintain the normal development of root stem-cell system by negatively regulating CK signaling (Muller and Sheen, 2008). *ARR16* and *ARR17* were proved to be key components in CK-mediated root hydrotropic response (Chang et al., 2019). Overexpression of *OsRR3* and *OsRR5* produced longer roots and more lateral roots compared with wild-type plants when treated with exogenous CK (Cheng et al., 2010). *OsRR2* is repressed by WUSCHEL-RELATED HOMEOBOX11 (WOX11), and they were involved in the processes of crown root elongation, lateral root primodium initiation, and root hair formation (Zhao et al., 2015; Cheng et al., 2016). In woody plants, overexpression of the type A RR *RcRR1* resulted in optimized root system with longer primary root and more lateral roots (Gao et al., 2013). In *Populus*, *PtRR13* negatively regulates the development of adventitious root (Ramirez-Carvajal et al., 2009).

Abiotic stress-responsive hormone ABA also plays an important role in mediating root growth and development. ABA enables its receptors to bind and sequester the type 2C protein phosphatases (PP2Cs), which induces auto-activation of class III SNF1-RELATED PROTEIN KINASE 2 (SnRK2s) to phosphorylate downstream transcription factors and regulates the expression of ABA-responsive gene (Sheard and Zheng, 2009; Wang et al., 2017). In *A. thaliana*, SnRK2.1, SnRK2.5, and SnRK2.9 were reported to control root growth under non-stress conditions, while SnRK2.4 and SnRK2.10 function mostly in root development under salt stress (Kawa et al., 2019). SnRK2.2 played an important role in root hydrotropism (Dietrich et al., 2017). In addition, NtSnRK2.2 positively regulated lateral root development to enhance salt tolerance (Liu et al., 2020). Overexpression of *TaSnRK2.4* or *TaSnRK2.7*, which are highly expressed in roots, resulted in better root system structure in *Arabidopsis* (Mao et al., 2010; Zhang et al., 2011).

The cross-talk between CK and ABA plays a vital role in root plasticity, and CK signaling probably act at downstream of ABA pathway (Basu et al., 2016; Rowe et al., 2016). In *Arabidopsis*, SnRK2.2, SnRK2.3, and SnRK2.6 directly interacted with phosphorylated ARR5 to mediate drought tolerance (Huang et al., 2018). However, there are few reports on the RRs in citrus and little knowledge on the interactions between RRs and SnRK2s.

*CcRR5*, a type A RR family member in citrus, was previously revealed to be involved in root morphogenesis, and its expression was upregulated at the early stages of PEG treatment (Zhang et al., 2022). In this study, the role of *CcRR5* was further explored to clarify the regulatory mechanism in the process of root development in citrus. Through stable genetic transformation mediated by *Agrobacterium tumefaciens*, *CcRR5* overexpressing and suppressed transgenic citrus plants were generated for further investigation of the function of *CcRR5* on growth and development of the roots. The relationships among CcRR5, CcRR14, and CcSnRK2s were analyzed in order to understand the molecular mechanism of root development in citrus.

## Materials and methods

### Plant materials and growth conditions

Volkamer (*Citrus limonia*) was used for cloning the gene for vector constructions. Trifoliate orange (*Poncirus trifoliata*) was used for genetic transformation. *Nicotiana benthamiana* was used for transient transformation and BiFC (bimolecular fluorescence complementation) assay. Experimental plants were grown in a greenhouse under a 16/8h light/dark photoperiod at 28°C.

### Subcellular localization of CcRR5

For subcellular localization, a coding sequence (CDS) fragment without termination codon of *CcRR5* was fused to the *Bam*HI/*Sac*I-digested Cam35S-GFP vector. The primers are shown in **Supplementary Table S1**. The fusion vector 35S::CcRR5-GFP and the green fluorescent protein (GFP) control were transformed into tobacco leaves (4–5 weeks old). GFP fluorescence was observed under a FV3000 confocal microscope (Olympus) as described by Du et al. (2023).

### GUS staining

The promoter of *CcRR5* was ligated to the *Bam*HI and *Hind*III sites of p1300GNGM-GUS vector to drive the *GUS* reporter gene. The CDS of *CcRR14* was cloned into the pFGC5941MDB3F-GN plasmid to obtain the 35S:CcRR14 recombinant plasmid. The combination was injected into tobacco leaves to measure the *GUS* expression as described by Li et al. (2020a).

The promoter of *CcRR5* was ligated to the *Bam*HI and *Hind*III sites of p1300GNGM-GUS vector and introduced into *P. trifoliata* plants. Roots, stems, and leaves were detached from transgenic plants and submerged in GUS dye solution at 37°C for 12h. The stained tissues were decolorized with 70% alcohol at 37°C for no less than 48h.

### Vector construction and genetic transformation

The CDS of *CcRR5* without termination codon was fused into the *Bam*HI/*Swa*I-digested pFGC5941MDB3F-GN vector to generate the 35S:CcRR5. For construction of RNAi vector, a 200–300 bp cDNA fragment of *CcRR5* was amplified and integrated into the pFGC5941MDB3F-GN vector. Subsequently, the constructs were introduced into *Agrobacterium tumefaciens* EHA105 by electroporation. Epicotyl explants of 1-month-old *P. trifoliata* were used to conduct *A. tumefaciens*–mediated transformation. The G418-containing MS medium was used to screen the transgenic plants. The positive transgenic citrus plants were identified by GFP fluorescence and genomic polymerase chain reaction (PCR). The expression of *CcRR5* in transformed plants was further confirmed by quantitative reverse transcription polymerase chain reaction (qRT-PCR) analysis.

### Morphological observation of the roots of transgenic plants

The shoots in the same length were cut from both transgenic and control plants of *P. trifoliata*. Cuttings were placed into a substance of mixed expanded perlite and vermiculite for rooting under 28°C. Rooted plantlets were grown in a greenhouse under a 16/8h light/dark photoperiod. Root indexes such as the length of root and number of root tips were determined.

### DNA, RNA extraction, and qRT-PCR

Citrus genomic DNA was extracted with a CTAB method (Zhang et al., 2020). The methods of RNA extraction, reverse transcription into cDNA, and qRT-PCR analysis were the same as described by Zhang et al. (2022).

### Bioinformatic analysis of SnRK2s in citrus

The protein sequences of citrus (*C. clementina*) were downloaded from Phytozome database (http://phytozome.jgi.doe.gov/pz/portal.html). Ten SNF1-related protein kinase 2s’ protein sequences of *Arabidopsis thaliana* (http://www.arabidopsis.org/) (AtSnRK2s) were used as reference sequences to identify the members of SnRK2 family in *C. clementina* (CcSnRK2s). The confirmation of SnRK2 family members, the gene structure and motifs analysis, the physicochemical properties, and the subcellular localization prediction of CcSnRK2s were performed as described by Zhang et al. (2022). Based on the Maximum Likelihood Method (MJ), the phylogenetic tree with protein sequences of SnRK2s from *A. thaliana* and citrus was constructed by using MEGA7.0 software (Kumar et al., 2016).

### Transactivation activity assay

To test the autoactivation of CcRR5, CcRR14, and CcSnRK2s, the vectors of pGBDT7-CcRR5, pGBDT7-CcRR14, and pGBDT7-CcSnRK2s were transferred into the cells of Y2HGold yeast strain, respectively. The yeast cells were cultured on synthetic defined (SD)/-Trp and SD/-Trp/-His/-Ade solid medium at 28°C for 2–3 days to observe the growth of the cells.

### Yeast two-hybrid

The CDS of *CcRR5* and *CcRR14* were inserted into the prey vector pGADT7, respectively, while the CDS of *CcSnRK2s* was cloned into the bait vector pGBKT7. Each pair of constructs was transformed using the lithium acetate method and then cultured on SD/-Trp/-Leu and SD/-Leu/-Trp/-His/-Ade with X-a-gal solid medium at 28°C.

### BiFC assays

The CDS of *CcRR14* and *CcSnRK2s* were cloned into pCV-cYFP and pCV-nYFP vectors, respectively. Recombinant plasmids were transformed into *A. tumefaciens* EHA105 cells, and each pair of combinations was co-transformed into tobacco leaves. YFP fluorescence was observed under a FV3000 confocal microscope (Olympus).

### Data analysis

Statistical analysis was performed by using the SPSS Statistics 17.0 based on Duncan’s test. Significant differences between different transgenic lines and wild-type plants were also subjected to Student’s *t*-test. The correlation analysis was conducted through bioinformatics (http://www.bioinformatics.com.cn/) with Pearson’s calculation method.

## Results

### Subcellular localization and GUS assay of CcRR5

**Figure 1:**
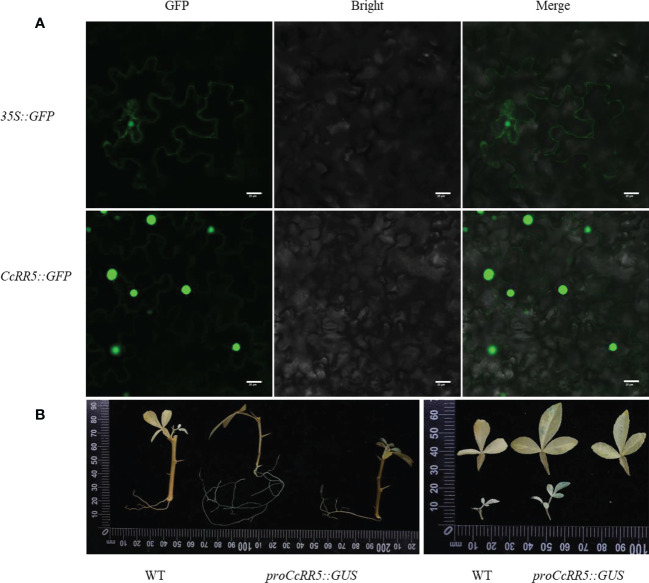
Subcellular localization and GUS assay of CcRR5. **(A)** Subcellular localization. **(B)** GUS assay. GFP, green fluorescent protein fluorescence; merge, merge of GFP fluorescence; bright field image. Bars = 20 µm. WT, wild type; *proCcRR5::GUS*, *proCcRR5::GUS* transgenic plants.

Transient expression assay was performed to validate subcellular localization of CcRR5 (**Figure 1A**). The 35S::GFP protein fluorescence signal was observed throughout the cell of tobacco leaves, while the 35S::CcRR5-GFP fusion protein fluorescence signal was observed only in the nuclear and cytomembrane, demonstrating that the protein of CcRR5 is localized in the nuclear and cytomembrane.

Previous studies have shown that the expression of *CcRR5* is not typical tissue specific, with higher expression in leaves and roots (Zhang et al., 2022). To further analyze the expression pattern of *CcRR5*, the *CcRR5*pro::GUS transgenic plants were generated in *P. trifoliata* background (**Figure 1B**). The GUS staining was strongly observed in both primary and lateral root tips of four-week-old plantlets,while weakly detected in old leaves.

### CcRR14 directly regulates the expression of *CcRR5* in citrus

**Figure 2:**
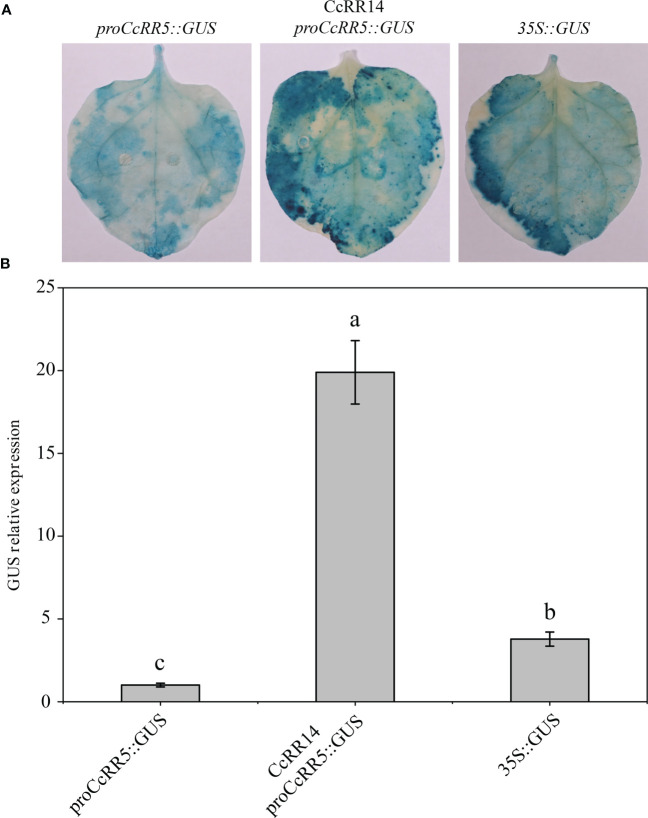
Protein–DNA interaction between CcRR14 and the *CcRR5* promoter. **(A)** GUS staining. **(B)** The expression level of *GUS*. Each error bar indicated the mean ± SD (*n* = 3). Different lowercase letters (a–c) mean significant difference, *p* < 0.05.

Previous study showed that the expressions of type A *CcRR5* and type B *CcRR14* in different citrus rootstocks were both correlated with the length of primary root (Zhang et al., 2022). In view of the relationship between type A and type B *RRs* in *Arabidopsis*, it could be speculated that CcRR14 may directly regulate the expression of *CcRR5*. The promoter of *CcRR5* was sequenced in this study (**Supplementary Figure S1**). The binding site of CcRR14 was found in the promoter of *CcRR5*, which indicated that *CcRR5* could be the direct target of CcRR14. To further examine whether CcRR14 is able to enhance the activity of the *CcRR5* promoter, we performed the transient expression assay with tobacco leaves. As shown in **Figure 2**, the expression of *GUS* was significantly higher in pro*CcRR5*::GUS plus CcRR14 combination (with strong staining) than that of the control (pro*CcRR5*::GUS, with weak staining). The results of promoter sequence analysis and GUS transient expression assays proved that CcRR14 can directly regulate the expression of *CcRR5* in citrus.

### Informatic analysis of SnRK2s in citrus

**Figure 3:**
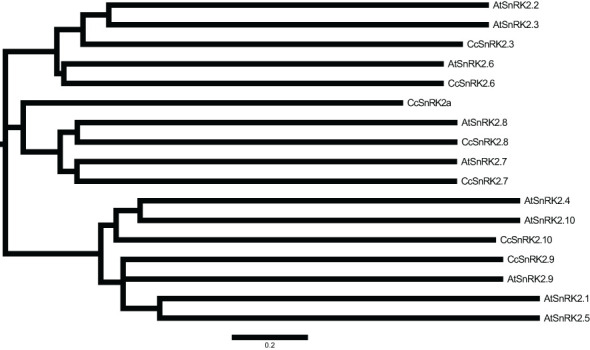
Phylogenetic tree of SnRK2 proteins from *Citrus clementina* and *Arabidopsis thaliana* CcSnRK2s: *C. clementina*; AtSnRK2s: *A. thaliana*. The scale bar was the distance scale.

**Figure 4:**
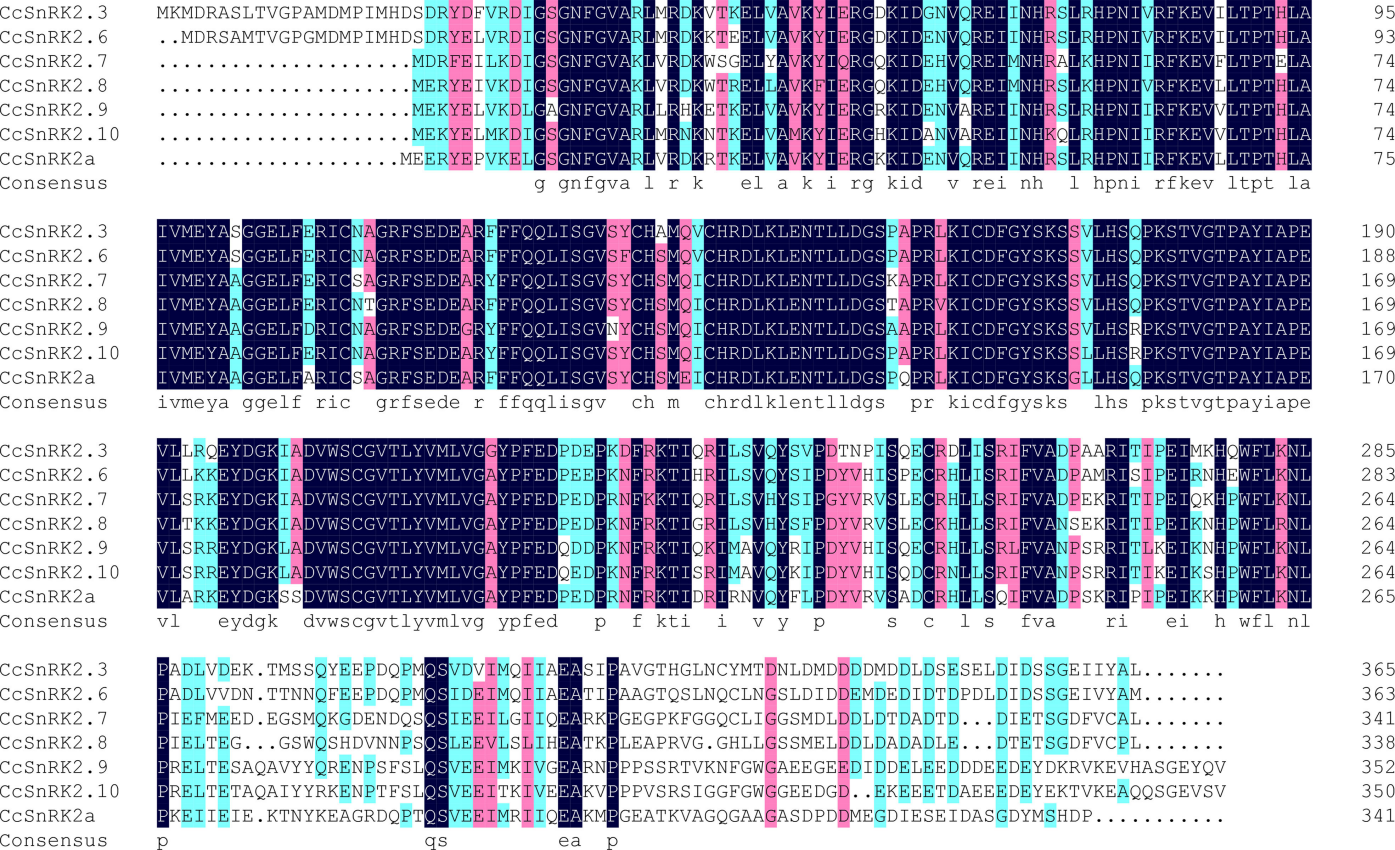
Amino acid sequence comparison of CcSnRK2s. Blueblack background: 100% similarity; blue: > 80% similarity; pink: > 50% similarity.

Expressions of *RR* genes in citrus were strongly induced by ABA, suggesting a close relationship between CcRRs and ABA signaling pathway (Zhang et al., 2022). SnRK2s, the key components of ABA signaling pathway, play important roles in the formation of root system. Referring to the interaction between ARR and AtSnRK2s in *Arabidopsis*, CcRRs might also interact with some members of SnRK2s in citrus. To comprehensively explore the potential interactions, analysis of the gene family is an essential method (Wang et al., 2015). Therefore, we performed genome-wide identification of SnRK2 family members in citrus. Seven SnRK2 members were identified from clementine genome after removing redundancy and alternative splicing sequences. The predicted physical and chemical properties of the seven members are shown in **Supplementary Table S2**. SnRK2s encode the proteins of 341–365 amino acids with molecular weights of 38.3–41.2 kD. They are hydrophilic proteins and rich in acidic amino acids, most of them are localized in the nucleus. In phylogenetic analysis (**Figure 3**), SnRK2s from *Arabidopsis* and citrus were divided into three groups. The members in citrus were termed based on their relationship with SnRK2s from *Arabidopsis*. Considering that Ciclev10020980m had no homolog in *Arabidopsis*, it was termed as CcSnRK2a alone. All the seven CcSnRK2s members possess the motif 1–7 (**Supplementary Figure S2**). Among them, CcSnRK2.3 and CcSnRK2.6 possessing the motif 8 were clustered in group 3, while CcSnRK2.9 and CcSnRK2.10 were placed into group 1 with motif 10. CcSnRK2a, CcSnRK2.7, and CcSnRK2.8 were classified in group 2. However, the motif 9 is only present in CcSnRK2.7 and CcSnRK2.8 but absent from CcSnRK2a. Sequence analysis showed that the N-terminal kinase domain of seven CcSnRK2s is relatively conserved, while the C-terminal domain, which plays a vital role in response to abiotic stress and ABA induction, is highly divergent (**Figure 4**).

### CcSnRK2s interact with both CcRR5 and CcRR14 in citrus

The RR interaction network in *Arabidopsis* was well studied previously, such as ARR involved in cytokinin and auxin signaling *via* AHP-ARR and ARR-PIN pathways (Hwang et al., 2012), and cytokinin and ABA signaling *via* ARR-AtSnRK2.2/2.3/2.6 pathway (Huang et al., 2018). However, little information on RR interaction network is available in woody plants. Therefore, it is necessary to explore whether the CcRR-CcSnRK2 network in citrus also works in the same way as that in *Arabidopsis* and how many members of the CcSnRK2 family can interact with CcRRs.

**Figure 5:**
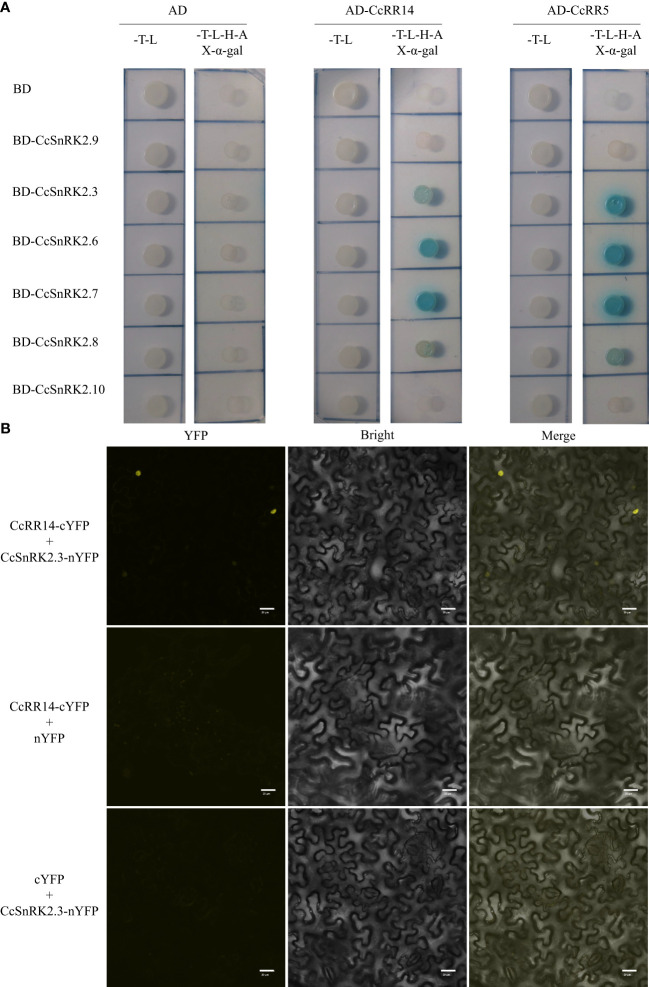
Interactions between CcRR5, CcRR14, and CcSnRK2s proteins. **(A)** Yeast two-hybrid assays between CcRR5, CcRR14, and CcSnRK2s. SD/-T-L, synthetic dropout medium lacking Trp and Leu. SD/-T-L-H-A, synthetic dropout medium lacking Trp, Leu, His, and Ade. **(B)** BiFC assays between CcRR14 and CcSnRK2.3. YFP, YFP fluorescence; merge, merge of YFP fluorescence; bright field image. Bars = 20 µm. .

To clarify these speculates, we studied the interactions between CcRR5 and CcSnRK2 subfamily proteins using yeast two-hybrid assay. First, the transcriptional activation of CcRR5, CcRR14, and CcSnRK2s was tested. The pGBKT7-CcRR5, pGBKT7-CcRR14, pGBKT7-CcSnRK2.3, pGBKT7-CcSnRK2.6, pGBKT7-CcSnRK2.7, pGBKT7-CcSnRK2.8, pGBKT7-CcSnRK2.9, and pGBKT7-CcSnRK2.10 fusion vectors (CcSnRK2a was not cloned from root sample of Volkamer) were constructed and transformed into Y2HGold yeast cells, respectively. As shown in **Supplementary Figure S3**, auto-activation was only observed in the yeast transformed with pGBKT7-CcRR14 in transactivation activity assay. In **Figure 5A**, CcRR5 showed significant interactions with CcSnRK2.3, CcSnRK2.6, CcSnRK2.7, and CcSnRK2.8 in Y2H assay. The results of yeast two-hybrid assay confirmed that CcRR14 also interacts with these four members of SnRK2. Those results were further confirmed by BiFC assay (**Figure 5B**, **Supplementary Figure S4**).

### Expressions of CcRR14 and SnRK2s partially corresponded to the expression of CcRR5 in the leaves of transgenic plants

**Figure 6:**
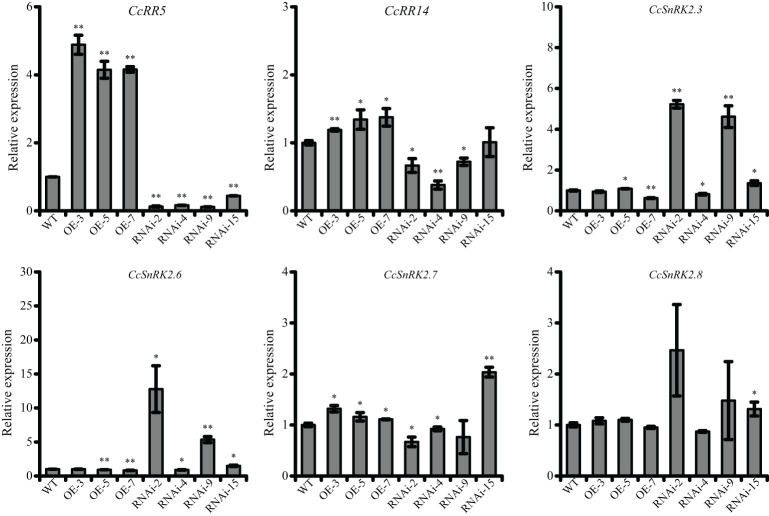
The expression level of *CcRR5*, *CcRR14*, and *CcSnRK2s* in transgenic plants. WT, wild type; OE, *CcRR5* overexpression plants; RNAi, *CcRR5* silence plants. Each error bar indicated the mean ± SD (*n* = 3). The asterisk indicated that the expression of *CcRRs* or *CcSnRK2s* in overexpression and silence plant was significantly different with that in the WT plants, *p < 0.05; **p < 0.01.

The 35S::CcRR5 and CcRR5-RNAi vectors were transformed into citrus rootstock *P. trifoliata*. Three overexpression lines (35S::CcRR5 OE-3, OE-5, and OE-7) and four RNAi-mediated gene knockdown lines (CcRR5-RNAi-2, RNAi-4, RNAi-9, and RNAi-15) were selected for further study (**Figure 6**). Compared with the control, the expression of *CcRR5* in overexpression lines increased more than three times, while that of in RNAi lines decreased to 0.13, 0.16, 0.12, and 0.44 times. The expressions of *CcRR14* and *CcSnRK2s* in transgenic plants were also analyzed. The expression of *CcRR14* was significantly upregulated in three overexpression plants and suppressed in three RNAi plants (RNAi-2, -4, and -9). The correlation between the expressions of these two genes was extremely significant (**Table 1**). The expressions of *CcSnRK2s* in transgenic plants were not consistent. The expressions of *CcSnRK2.3* and *CcSnRK2.6* in three RNAi plants (RNAi-2, -9, and -15) were significantly upregulated. The correlation between the expression of *CcSnRK2.3* and *CcRR5* was extremely significant, while the correlation between the expression of *CcSnRK2.6* and *CcRR5* was significant. Even though the expression of *CcSnRK2.7* was upregulated in overexpression plants and corresponded with the expression of *CcRR5*, the correlation was not significant.

**Table 1:** The correlation between *CcRR5* and *CcSnRK2s* expression in transgenic plants.

	*CcRR5* and *CcRR14*	*CcRR5* and *CcSnRK2.3*	*CcRR5* and *CcSnRK2.6*	*CcRR5* and *CcSnRK2.7*	*CcRR5* and *CcSnRK2.8*
*r*	0.801	−0.540	−0.456	0.183	−0.347
*p* value	0.000**	0.006**	0.025*	0.391	0.096

*p < 0.05; **p < 0.01.

### Overexpression of CcRR5 promotes the root growth in citrus

**Figure 7:**
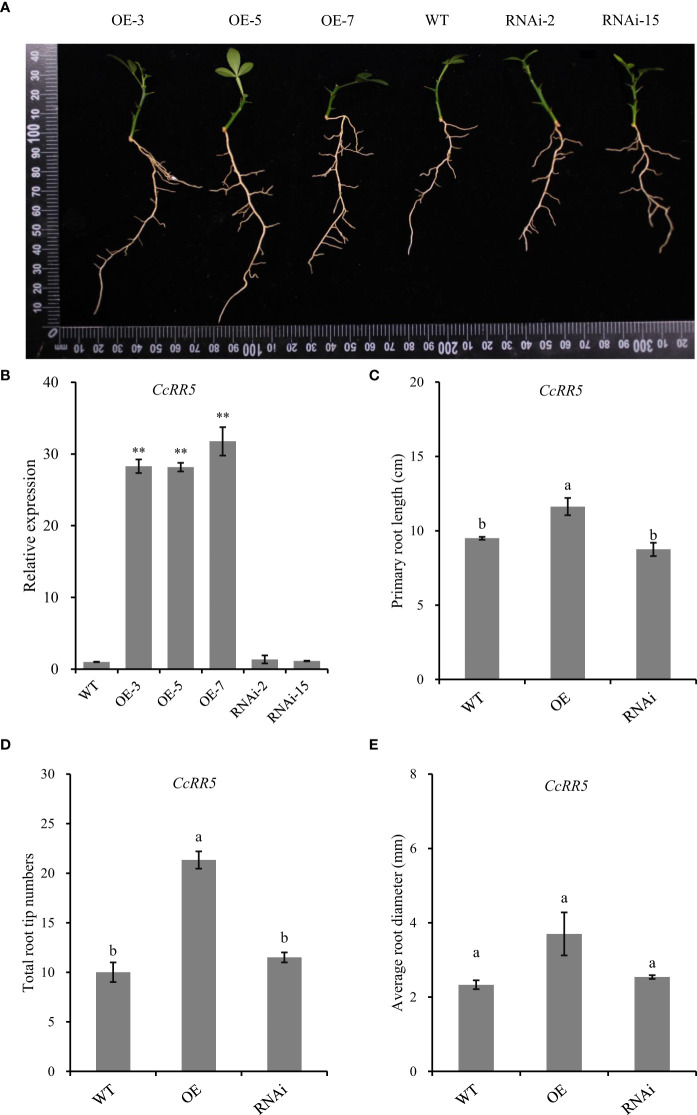
Functional analysis of *CcRR5* in citrus. **(A)** Morphological observations of roots in *CcRR5* overexpression (CcRR5-OE) and *CcRR5* RNA interference (CcRR5-RNAi) transgenic plants. **(B)** Relative expression of CcRR5 in transgenic plants, **(C)** primary root length of plantlet, **(D)** total root tip numbers of plantlet, and **(E)** average root diameter of plantlet. The error bars in **(B)** indicated the mean ± SD (*n* = 3). The asterisk indicated that the expression of *CcRR5* in overexpression and silence plants was significantly different with that in the WT plants. **p < 0.01. The error bars in **(C–E)** indicated the mean ± SE. Means followed by different letters (a, b) indicate significantly different at *p* < 0.05.

Phenotypes of different transgenic plants were shown in the **Figure 7A**. The qRT-PCR analysis of self-rooted plants showed that compared with the control, the expression of *CcRR5* was significantly upregulated in the roots of the overexpressed plants but had no difference in the roots of the silenced plants (**Figure 7B**). In *CcRR5* overexpression lines, the primary root lengths were significantly longer than those of the control plants (**Figure 7C**). Total numbers of root tips of CcRR5-OE lines were also significantly greater than those of the control plants (**Figure 7D**). However, silence of *CcRR5* did not affect the growth of root in RNAi plants.

**Figure 8:**
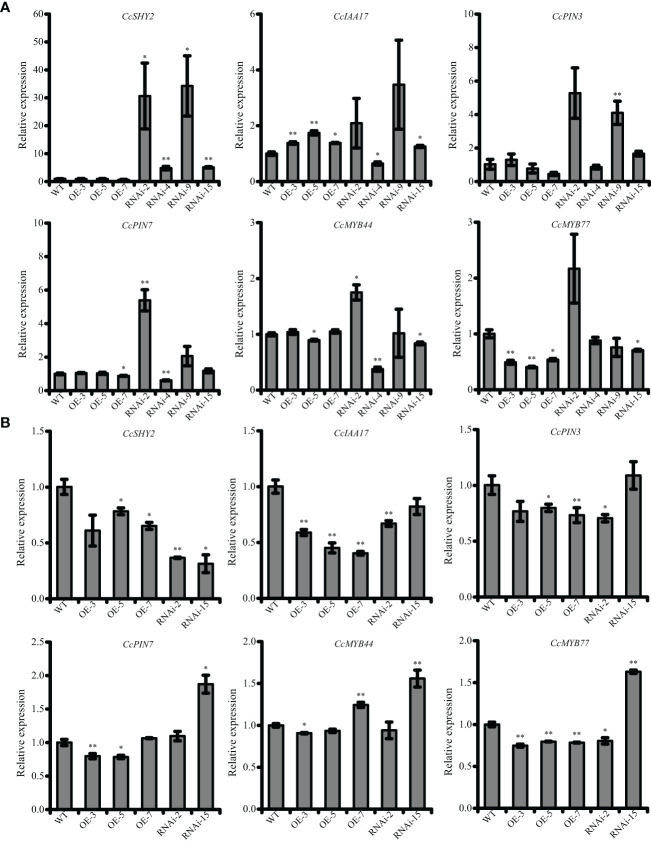
The expression levels of root-related genes in the leaves **(A)** and roots **(B)** of *CcRR5* transgenic plants. WT, wild type; OE, *CcRR5* overexpression plants; RNAi, *CcRR5* silence plants. Each error bar indicated the mean ± SD (*n* = 3). The asterisk indicated that the expression of genes in overexpression and silence plant was significantly different with that in the WT plants. *p < 0.05; **p < 0.01.

**Figure 9:**
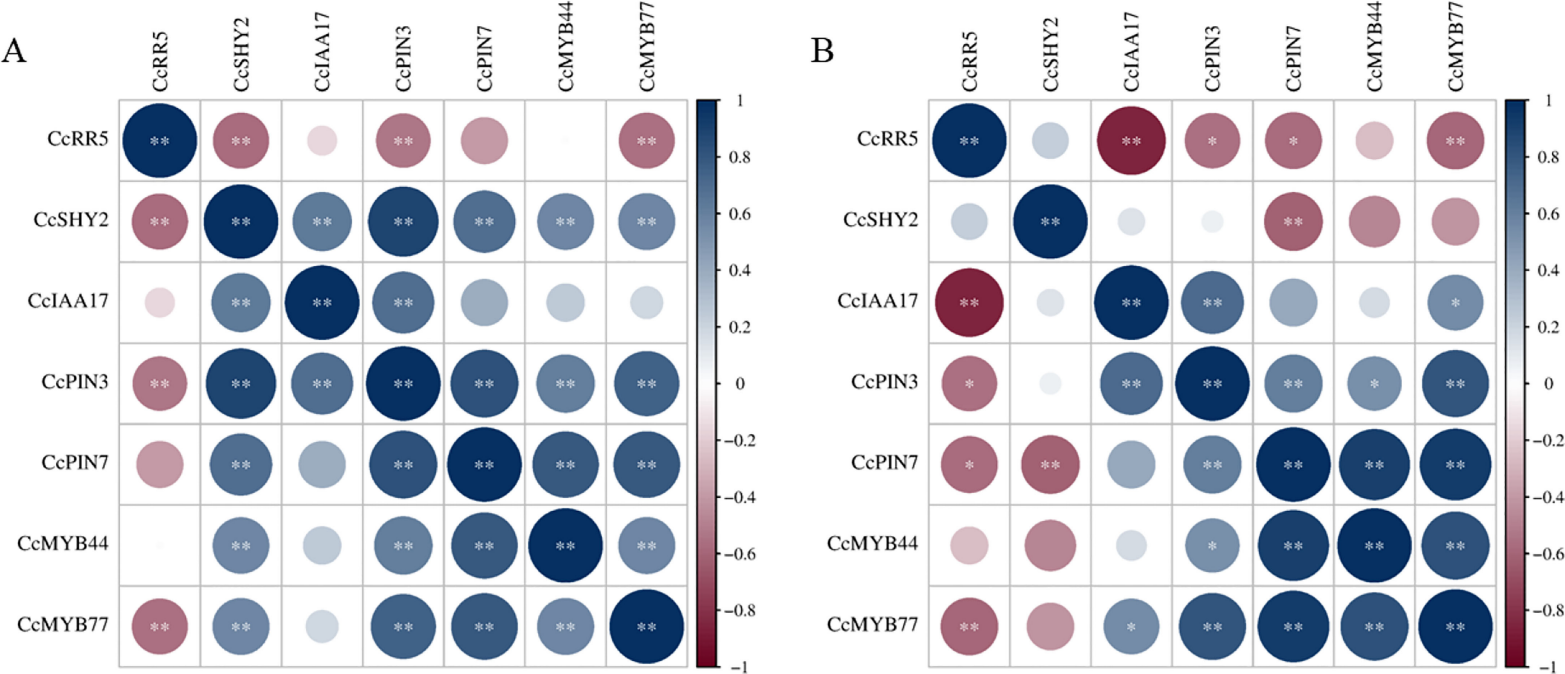
The correlation between expressions of *CcRR5* and root-related genes in the leaves **(A)** and roots **(B)** of *CcRR5* transgenic plants. The color bar represented the range of correlation coefficient (*r*). *p < 0.05; **p < 0.01.

To further investigate the involvement of *CcRR5* in root development, the expression levels of some genes might relate to root growth and development were determined by qRT-PCR (**Figure 8**). Results showed that the expression of *CcMYB77* in the roots and leaves of CcRR5-OE lines was significantly suppressed. The correlation between the expression of *CcMYB77* and *CcRR5* was extremely significant in both roots and leaves (**Figure 9**). The expression of *CcIAA17* showed the opposite trend with that of *CcMYB77* in leaves, but the same trend in roots, which was extremely negatively corresponded to the expression of *CcRR5*. In addition, the expressions of *CcPIN3* and *CcSHY2* were also extremely significantly correlated to the expression of *CcRR5* in leaves, while the expressions of *CcPIN3* and *CcPIN7* were significantly correlated to the expression of *CcRR5* in roots. These results suggested that *CcRR5* involved in the regulation of root growth and played a positive role in root morphogenesis.

## Discussion

### Interactions between CcRR5, CcRR14 and CcSnRK2s

It was reported that type B ARR family members serve as transcriptional regulators of type A *ARRs.* They usually bind to the cytokinin response motif or extended motif (Tajima et al., 2004; Ramireddy et al., 2013). Both type A *ARR5* and *ARR15* are direct targets of the transcriptional type B RR, ARR2 (Che et al., 2008). Interestingly, the expression of type B *CcRR14*, in the same group of a phylogenic tree with *ARR1* and *ARR2*, was significantly correlated with the expression of type A *CcRR5* in our previously study (Zhang et al., 2022), suggesting that CcRR14 might regulate the expression of *CcRR5*. In this study, we identified that promoter of *CcRR5* contains several binding elements of type B RRs. In *CcRR5* transgenic plants, the expression of *CcRR14* was significantly correlated to the expression of *CcRR5.* In GUS assay, the activity of *CcRR5* promoter was enhanced by *CcRR14*. All those findings confirmed the hypothesis that CcRR14 directly regulates the expression of *CcRR5*.

ABA signaling cascade serves as crucial factor in aspects of plant growth and development (Wang et al., 2017). Currently, stress response mediated by SnRK2s pathway has been widely studied in model plants (Miao et al., 2021). Here, seven SnRK2 members were identified from *C. clementina*, which was consistent with the number of SnRK2s in *P. trifoliate*, but less than that of in *Arabidopsis*, maize, cotton, and apple, due to the retraction or diversification of gene and the duplication of whole-genome *(* Huai et al., 2008 *;* Liu et al., 2017 *;* Shao et al., 2014 *;* Song et al., 2022 *)*. The Group III members of SnRK2 were reported to strongly interact with ARR1 and ARR5 *in Arabidopsis* (Huang et al., 2018). In this research, we verified the interaction between CcRRs and SnRK2 family members and had some new discoveries. In addition to CcSnRK2.3 and CcSnRK2.6, group II members CcSnRK2.7 and CcSnRK2.8, homologous to AtSnRK2.7 and AtSnRK2.8, can also interact with CcRR5 and CcRR14. It can be assumed that CcRR5, CcRR14, and CcSnRK2s in citrus should work in the similar way as that in *Arabidopsis* (Huang et al., 2018), that the type B CcRR14 positively regulates the cytokinin signaling and inhibits the activity of CcSnRK2s, while the type A *CcRR5*, upregulated by CcRR14 in a negative feedback loop, negatively regulates the cytokinin signaling and be phosphorylated by CcSnRK2s to regulate the expression of ABA-responsive genes.

### *CcRR5* acts as a positive regulator of primary root growth

*CcRR5*, a type A cytokinin RR in citrus, is homologous to *ARR5*/*6*/*7*/*15*, and its expression was significantly correlated with primary root length (Zhang et al., 2022). In this research, overexpression of *CcRR5* in citrus resulted longer primary root and greater number of lateral roots compared to the control, which is in agree with the findings of Kiba et al. (2003) and To et al. (2007) in *Arabidopsis*. Recent studies have reported that ARR1 is a negative regulator of primary root growth under aluminum and salt stresses (Yang et al., 2017b; Yan et al., 2021). This suggested that CcRR14 might directly bind to the promoter of *CcRR5* and regulate its expression to affect the elongation of citrus roots in response to abiotic stresses. Overexpression of *AtSnRK2.8*, a root-specific protein kinase, increased the tolerance of drought (Umezawa et al., 2004). AtSnRK2.4 and AtSnRK2.10 can maintain root growth under salt stress (McLoughlin et al., 2012). Better root architecture with enhanced salt-stress tolerance was also achieved in tobacco by overexpression of *NtSnRK2.2*, a group II member in *Nicotiana tabacum* L. (Liu et al., 2020). The interactions between CcSnRK2s and CcRRs were further confirmed that CcRR5 contributed to the root growth. More evidences have been observed that expressions of auxin-related genes such as *PINFORMEDs* (*PINs*), auxin signal inhibitor *SHY2*, and auxin/indole-3-acetic acid *IAA17* were significantly correlated to the expression of *CcRR5*. Those genes are the key factors in regulating root meristem size and adventitious root formation in *Arabidopsis* (Hwang et al., 2012; Liu et al., 2015; Lakehal et al., 2019). The expression of *MYB77* was significantly decreased in CcRR5-overexpression citrus plants, which was reported to be a negative factor of primary root growth strongly expressed in lateral and primary roots in *Arabidopsis* (Shin et al., 2007). All those findings indicated that *CcRR5* is involved in formation of root system architecture, which might be regulated by some genes involved in other hormone signal pathways.

## Conclusions

The type A RR CcRR5 is mainly localized in the nucleus and expressed in root tips and young leaves in citrus. Phenotypic comparison and expression analysis of transgenic citrus plants indicated that *CcRR5* could enhance the growth of roots and formation of lateral roots. The promoter activity of *CcRR5* can be enhanced by CcRR14. They both can interact with CcSnRK2s.

## Data availability statement

The original contributions presented in the study are included in the article/**Supplementary Material**. Further inquiries can be directed to the corresponding authors.

## Author contributions

SZ and XZ conceived the project. MZ, SZ and XZ designed the experiments. MZ, FW, ZH, XW, QY and JF performed the experiments. MZ, SZ and XZ wrote and revised the manuscript. All authors contributed to the article and approved the submitted version.
