# Supplementary material for: CcRR5 interacts with CcRR14 and CcSnRK2s to regulate the root development in citrus
Source: Front Plant Sci. 2023 Apr 17;14:1170825. doi: 10.3389/fpls.2023.1170825 (PMC10150009; doi:10.3389/fpls.2023.1170825)
Supplement: Supplementary file 1 [file DataSheet_1.docx]

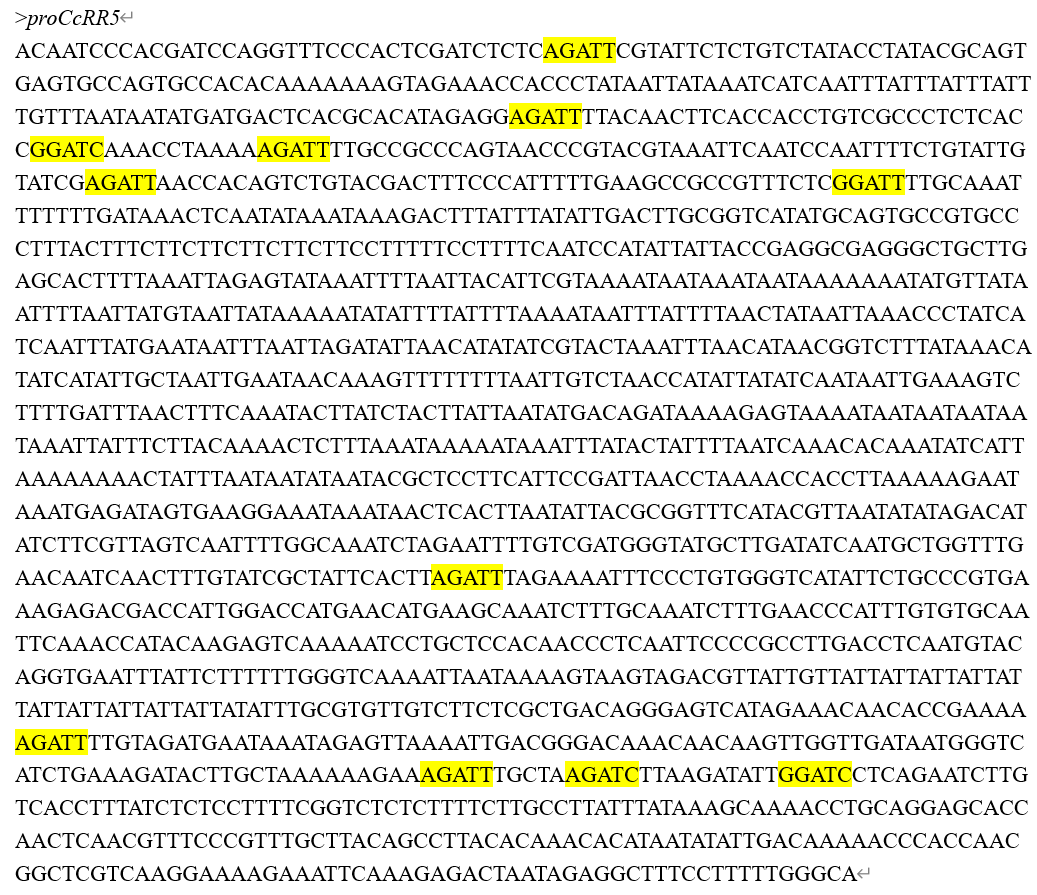


Figure S1 Promoter sequence of CcRR5.

The highlighted bases are cytokinin response motifs (CRM): 5’-(A/G)GAT(T/C)-3’.


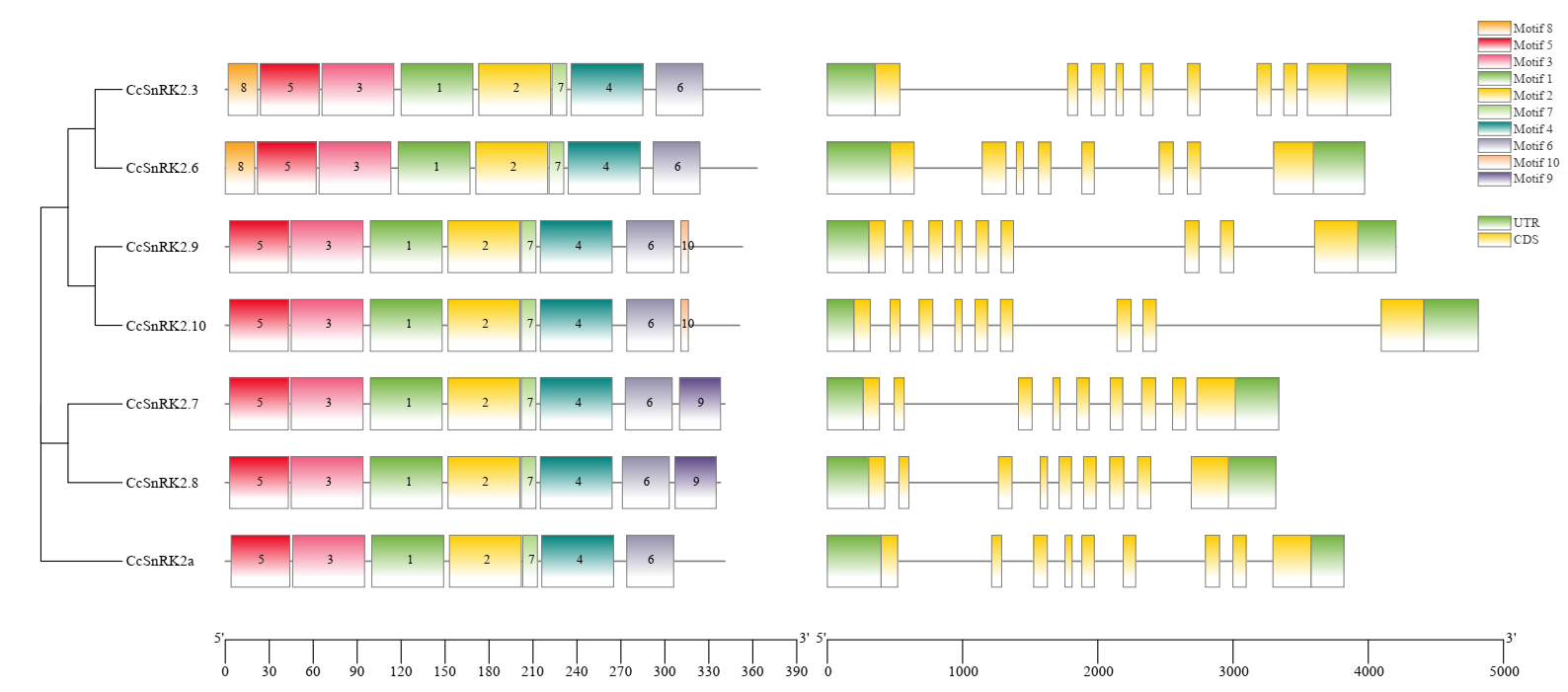


Figure S2 Phylogenetic relationship, conserved domains and exon-intron structures of CcSnRK2s.

Left: The phylogenetic tree constructed with the N-J method with MEGA 7. Middle: Distribution of conserved domains of CcSnRK2s. The relative positions of each domain are shown by colored bars. Right: The exons, introns and untranslated regions (UTRs) are represented by yellow rectangles, black lines and green rectangles, respectively.


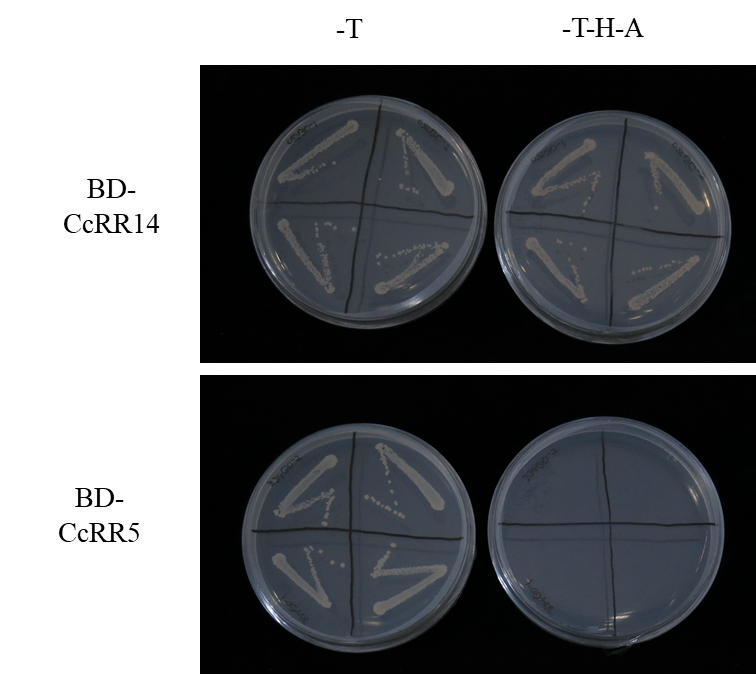


Figure S3 Identification of the transcriptional activation of CcRR5 and CcRR14.

SD/-T, synthetic dropout medium lacking Trp. SD/-T-H-A, synthetic dropout medium lacking Trp, His, and Ade.


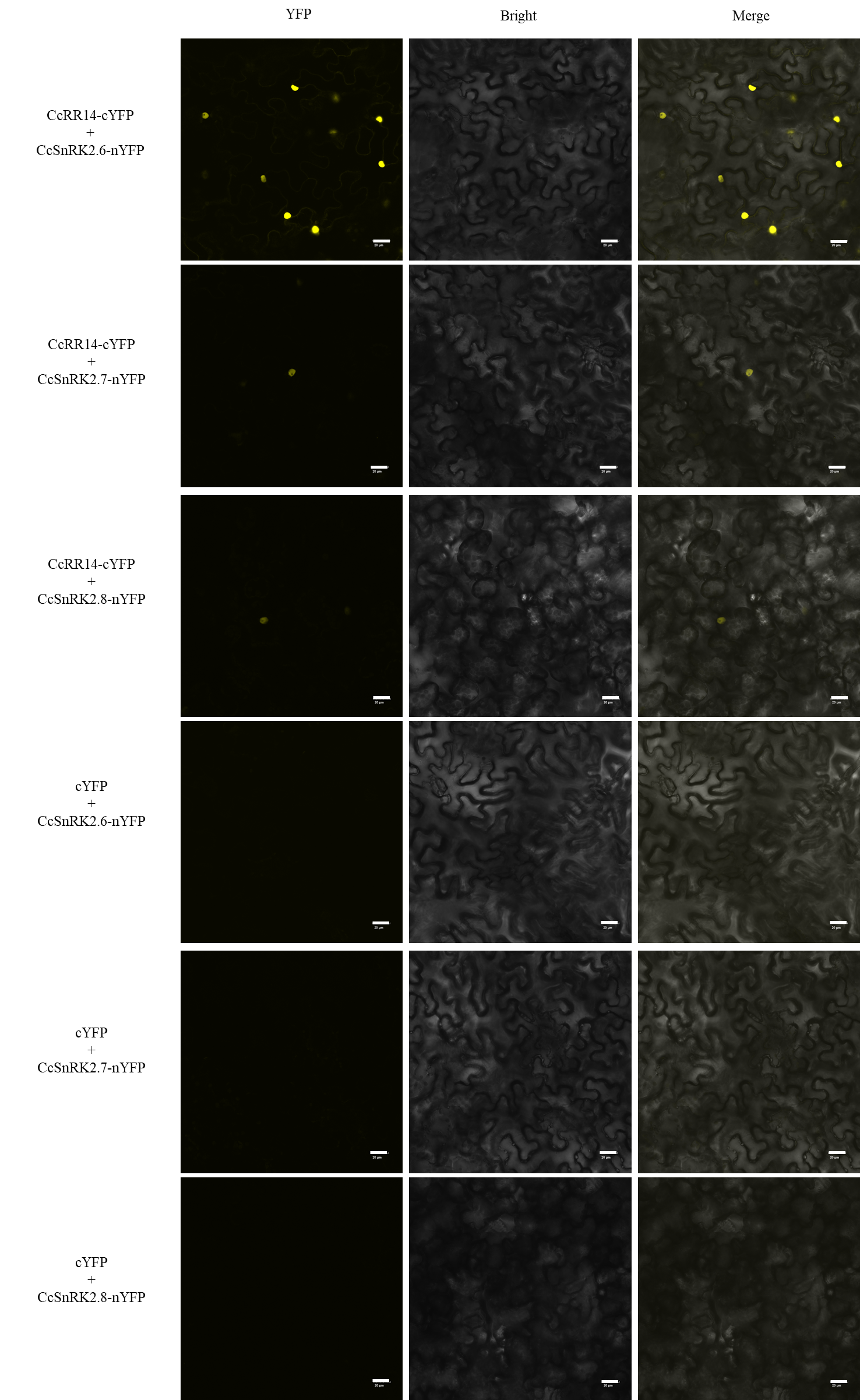


Figure S4 BiFC assay of the interaction between CcRR14 and CcSnRK2s.

YFP, YFP fluorescence; Merge, merge of YFP fluorescence, and Bright field image. Bars = 20 µm.

Table S1 Primers used for real-time quantitative PCR, subcellular localization, GUS assay, Y2H, BiFC assay, overexpression and RNAi genetic transformation

| Gene Name | Forward Primer (5’to 3’) | Reverse Primer (5’to 3’) |
| --- | --- | --- |
|  | **qRT-PCR** |  |
| *CcRR5* | CATGCCTGGGATGACTGGAT | ATTCCTCTGCCCCTTCTTCC |
| *CcRR14* | GGTCAGCATCTCAATAGCCTTC | CCATCCTGCTGCTGTTTCAG |
| *CcSHY2* | GTAATGAGAGGGACCCAATTCA | GTACAGTTCATCGTTCAAAGCA |
| *CcIAA17* | TGAAACGGCAGCATTTGTGA | AGTCGCCATCCTTGTCTTCA |
| *CcPIN3* | CACTTACTCCAGCTTAATTGGC | TGGAGATGGACTTTGCTACAAT |
| *CcPIN7* | GACGGAATCAGAAACGGACG | TGTAAAGGAGCCGGAGTGTT |
| *CcMYB44* | CAAATCAATTCCCGGAAGATCG | TACGTTTTAAGGTCGAGTTCCA |
| *CcMYB77* | AGTGAGAAACTACATGTCTGGG | TTAATCCCGATACGCTTAACCA |
| *CcSnRK2.3* | CCCAGCTCCTCGGTTAAAGA | TATCCTCCCACCAGCATCAC |
| *CcSnRK2.6* | AGAGCCAAAGAACTTCCGCA | ATCCTCATTGCAGGGTCAGC |
| *CcSnRK2.7* | AGTCCTCAGTGTTGCACTCG | TGGACACTCAGAATCCGCTG |
| *CcSnRK2.8* | GCTGCTGGAGGAGAACTGTT | TTAACACGTGGAGCAGTGCT |
| *CcSnRK2.9* | AACAGTTGGAACTCCGGCTTAT | GGGATTCTGTACTGAACTGCCA |
| *CcSnRK2.10* | CATACGCACACACATATACAGC | TTGTTTCTCATAAGCCTAGCCA |
| *CcSnRK2a* | TGCTGCTGGTGGTGAACT | TCAGCCGTGGTTGTGGAC |
| *Ccβactin* | CCGACCGTATGAGCAAGGAAA | TTCCTGTGGACAATGGATGGA |
|  | **subcellular localization** |  |
| *CcRR5sub* | agaacacgggggacgagctcATGGCAACAGTGGCTGAATG | cgactctagaggatccGGCAAGTAATTTAGGTCGCTTG |
|  | **pro-GUS, over-expression and RNAi genetic transformation** |  |
| *CcRR5pro* | cgacggccagtgccaagcttACAATCCCACGATCCAGGTT | ggactgaccacccggggatccTGCCCAAAAAGGAAAGCCT |
| *OE-CcRR5* | gcccaatcgatgatttaaatATGGCAACAGTGGCTGAATG | ctctagactcacctaggatccGGCAAGTAATTTAGGTCGCTTG |
| *RNAi-CcRR5* | tctagaatttaaatGTGACGGCTGTGGAGAGTGG | ggatccatttaaatGCCATTTTCCTGTGGTTCTCC |
|  | **Y2H** |  |
| *AD-CcRR5* | tggccatggaggccagtgaattcATGGCAACAGTGGCTGAATG | tgcagctcgagctcgatggatccGGCAAGTAATTTAGGTCGCTTG |
| *BD-CcRR5* | atatggccatggaggccgaattcATGGCAACAGTGGCTGAATG | ggccgctgcaggtcgacggatccGGCAAGTAATTTAGGTCGCTTG |
| *AD-CcRR14* | tggccatggaggccagtgaattcATGAATCTCAATAACGATAAAGGATCC | tgcagctcgagctcgatggatccTATCACGGGAATATTGTCCAGGG |
| *BD-CcRR14* | atatggccatggaggccgaattcATGAATCTCAATAACGATAAAGGATCC | ggccgctgcaggtcgacggatccTATCACGGGAATATTGTCCAGGG |
| *BD-CcSnRK2.3* | atatggccatggaggccgaattcATGAAAATGGATCGCGCAT | ggccgctgcaggtcgacggatccCCGCAACGCATATATTATCTCC |
| *BD-CcSnRK2.6* | atatggccatggaggccgaattcATGGATCGATCCGCGATGA | ggccgctgcaggtcgacggatccCCGCATAGCATATACAATCTCCC |
| *BD-CcSnRK2.7* | atatggccatggaggccgaattcATGGACCGTTTCGAGATTCTG | ggccgctgcaggtcgacggatccTCATAATGCACAGACAAAATCGC |
| *BD-CcSnRK2.8* | atatggccatggaggccgaattcATGGAGAGATATGAGATTGTGAAGG | ggccgctgcaggtcgacggatccTCACAATGGGCACACAAAATC |
| *BD-CcSnRK2.9* | atatggccatggaggccgaattcATGGAGAAATATGAATTGGTGAAGG | ggccgctgcaggtcgacggatccTTAATTGACTTGATATTCTCCACTTGC |
| *BD-CcSnRK2.10* | atatggccatggaggccgaattcATGGAGAAGTATGAGCTTATGAAGG | ggccgctgcaggtcgacggatccTTAACTGACGCTAACTTCTCCACT |
|  | **BiFC** |  |
| *c-CcRR14* | gagctgtacaagtccggagtcgacATGAATCTCAATAACGATAAAGGATCC | gaattcgagctcgcctggggatccCTACACGGGAATATTGTCCAGG |
| *n-CcSnRK2.3* | aacatcgaggactccggagtcgacATGAAAATGGATCGCGCAT | gaattcgagctcgcctggggatccTTACAACGCATATATTATCTCCCC |
| *n-CcSnRK2.6* | aacatcgaggactccggagtcgacATGGATCGATCCGCGATGA | gaattcgagctcgcctggggatccTCACATAGCATATACAATCTCCCC |
| *n-CcSnRK2.7* | aacatcgaggactccggagtcgacATGGACCGTTTCGAGATTCTG | gaattcgagctcgcctggggatccTCATAATGCACAGACAAAATCGC |
| *n-CcSnRK2.8* | aacatcgaggactccggagtcgacATGGAGAGATATGAGATTGTGAAGG | gaattcgagctcgcctggggatccTCACAATGGGCACACAAAATC |
| *n-CcSnRK2.9* | aacatcgaggactccggagtcgacATGGAGAAATATGAATTGGTGAAGG | gaattcgagctcgcctggggatccTTAATTGACTTGATATTCTCCACTTGC |
| *n-CcSnRK2.10* | aacatcgaggactccggagtcgacATGGAGAAGTATGAGCTTATGAAGG | gaattcgagctcgcctggggatccTTAACTGACGCTAACTTCTCCACT |

Table S2 Physicochemical properties and subcellular localization prediction of CcSnRK2s.

| Family  member | Genome  code | Number of amino acids (aa) | Molecular weight (Da) | Theoretical pI | Grand average of hydropathicity (GRAVY) | Subcellular localization |
| --- | --- | --- | --- | --- | --- | --- |
| CcSnRK2.3 | Ciclev10025916m | 365 | 41228.93 | 4.76 | -0.269 | Nuclear |
| CcSnRK2.6 | Ciclev10028703m | 363 | 41113.73 | 4.83 | -0.294 | Nuclear |
| CcSnRK2.7 | Ciclev10026004m | 341 | 38668.06 | 5.25 | -0.355 | Cytoplasmic |
| CcSnRK2.8 | Ciclev10001754m | 338 | 38321.72 | 5.74 | -0.293 | Nuclear |
| CcSnRK2.9 | Ciclev10025965m | 353 | 40689.07 | 5.85 | -0.569 | Nuclear |
| CcSnRK2.10 | Ciclev10001672m | 351 | 40121.66 | 6.06 | -0.51 | Nuclear; Cytoplasmic |
| CcSnRK2a | Ciclev10020980m | 341 | 38708.11 | 5.92 | -0.489 | Cytoplasmic |
